# Transcriptomic Analysis of Domestic Ducks’ Proventriculus Infected with *Eustrongylides tubifex* (Nitzsch 1819) Jägerskiöld 1909

**DOI:** 10.3390/vetsci13050487

**Published:** 2026-05-18

**Authors:** Cuilan Hao, Yujiao Bai, Shenzhen Xia, Cheng Yue, Adili Abudu, Jianyong Hu, Wei Guo

**Affiliations:** 1 College of Veterinary Medicine, Xinjiang Agricultural University, Urumqi 830052, China; haocuilan@126.com (C.H.); 19049039676@163.com (Y.B.); 13639920348@163.com (S.X.); yuechengxnd@aliyun.com (C.Y.); 2 Xinjiang Key Laboratory for Ecological Adaptation and Evolution of Extreme Environment Organisms, College of Life Sciences, Xinjiang Agricultural University, Urumqi 830052, China; 3 Xinjiang Key Laboratory of New Drug Research and Development for Herbivorous, Urumqi 830052, China; 4 Xinjiang Regional Key Laboratory of Clinical Veterinary Medicine Research, Urumqi 830052, China; 5 Xinjiang Uygur Autonomous Region Fisheries Development Center, Xinjiang Uygur Autonomous Region Fisheries Science Research Institute, Urumqi 830099, China; xjadili@163.com (A.A.); hujianyong2007@163.com (J.H.)

**Keywords:** domestic ducks, *Eustrongylides tubifex*, transcriptomics, host–parasite interaction

## Abstract

Gastric nematodes can cause severe diarrheal diseases of waterfowl and threaten waterfowl health and productivity. The immune response mechanism of the host against this parasite is unclear. We investigated the transcriptomic changes, immune related pathways and adaptive molecular responses in infected ducks. RNA sequencing of the gastric tissues of infected and control ducks was followed by differential expression using DESeq2, and functional enrichment and protein–protein interaction networks were constructed. There were 1983 differentially expressed genes (DEGs), including 1317 upregulated genes and 666 downregulated genes. GO and KEGG analyses showed considerable enrichment in immune response, extracellular matrix organization, and chemotaxis and cytokine-mediated signaling pathways, suggesting systemic immune activation and tissue remodeling. The protein–protein interaction network indicated several hub genes that might be central to host defense. Validation by RT-qPCR showed consistent expression trends for some DEGs. These results systematically outline the transcriptomic landscape of ducks infected with *Eustrongylides tubifex*, offering new insights into host–parasite interactions and immune modulation. The results enhance our understanding of anti-helminth immunity in waterfowl and may inform strategies for disease control in poultry production.

## Simple Summary

Gastric nematodes can cause severe diarrheal diseases of waterfowl, harming waterfowl health and farm productivity. To better understand how ducks defend against gastric nematodes, we investigated the transcriptomic changes, immune-related pathways and adaptive molecular responses in infected ducks. By comparing stomach tissues from infected groups and constructed groups in ducks, we identified 1983 differentially expressed genes (DEGs), including 1317 upregulated genes and 666 downregulated genes. Many genes were involved in immune responses and tissue repair. Key genes linked to defense were also identified. These results reveal how ducks respond to gastric nematodes at the molecular level and offer new clues for improving disease control and waterfowl health management.

## 1. Introduction

Gastrointestinal nematodes are an important threat to poultry health and productivity and cause substantial losses due to poor growth, poor feed conversion, and higher mortality in poultry production [1]. Domestic ducks (*Anas platyrhynchos domesticus*) are highly susceptible to a variety of helminth parasites in free-range or semi-intensive systems. The genus *Eustrongylides* belonging to the family Dioctophymidae, order Enoplida, is a common parasite condition found in fishes and waterfowl [2], and it exhibits a complex life cycle. The first intermediate host of *Eustrongylides* is Oligochaeta, the second intermediate host is fishes, and its definitive hosts are ducks and carnivorous birds [3]. Anand S. Rautela provided the classification description of the species in genus *Eustrongylides* [4]. Currently, *Eustrongylides* is recognized to comprise three valid species: *Eustrongylides tubifex* (Nitzsch 1819) Jägerskiöld 1909, *E. ignotus* Jägerskiöld, 1909, and *E. excisus* Jägerskiöld, 1909. *E. tubifex* is a risky parasite leading to tissue inflammation, ulceration and poor digestion [5,6,7,8], but these effects are not severe.

High-throughput transcriptomic technologies have revolutionized host–parasite interactions and provide a comprehensive and unbiased profile of gene expression changes after infection. RNA-Seq is adopted to analyze the mechanisms underlying immune responses and disease processes in hosts with different helminths in the field of veterinary parasitology [9,10,11,12,13,14,15]. RNA-Seq of infections with *Ascaridia galli* and *Heterakis gallinarum* shows significant modulation of immune pathways (Toll-like receptor, cytokine–cytokine receptor interactions, T-helper cell differentiation pathways in chickens) [16,17,18,19]. RNA-Seq studies on chickens with nematodes such as *Ascaridia galli* and *Heterakis gallinarum* show significant modulation of the immune response of the immune arms, both innate and adaptive arms, leading to the upregulation of genes that encode proinflammatory cytokines, chemokines and antigen presentation molecules. In mammals and other birds, helminth infections induce major remodelulation of the local tissue environment involving extracellular matrix structure, wound-healing processes, and metabolic reprogramming [20,21]. For instance, infections often trigger a type 2 immune response related to alternative activation of macrophages and the involvement of specific interleukins (e.g., IL-4, IL-13), which can contribute to both parasite expulsion and tissue repair [22].

There are still some questions concerning the specific transcriptional state of ducks infected with gastric nematodes, even with this progress in related species and helminth systems. Most transcriptomics studies focus on chickens or mammalian models, but the molecular responses of waterfowl with different immunological and physiological features are still unclear [23]. The early host response in gastric tissues, the precise identity of key regulatory genes (hub genes) controlling the interaction network, and possible interaction between immune activation and systemic metabolic changes are still not clear in ducks [24,25]. Bulk RNA-Seq provides a global overview, but mask cell type-specific responses in gastric tissues (e.g., epithelial cells, immune cells and fibroblasts) are unclear and may play unique role in host response [26].

We conducted a complete transcriptomic analysis of gastric tissues from domestic ducks infected with *E*. *tubifex* and applied differential expression analysis, gene ontology (GO) and Kyoto Encyclopedia of Genes and Genomes (KEGG) pathway enrichment, GSEA and PPI network construction to gain systems-level knowledge of the host response. The robustness of the transcriptomic data was validated using quantitative real-time PCR (RT-qPCR). Our results will provide valuable insight on nematodiasis in ducks, identify potential genes for disease resistance, and lead to novel intervention strategies against parasitic worms in poultry.

## 2. Materials and Methods

### 2.1. Animals and Infection Protocol

Ten 45- to 60-day-old female domestic ducks (*Tadorna*) (*Anas platyrhynchos domesticus*) weighing 1.39 ± 0.10 kg and with no history of parasitic infection were procured from a local commercial market. Upon arrival, all domestic ducks were housed (24 ± 4 °C, 12 h light/dark cycle) in standard iron cages within a controlled animal facility. They were offered with ad libitum access to water and a standard commercial duck diet twice daily. After a two-week acclimatization period, the domestic ducks were divided randomly into an experimental infection group or an uninfected control group (both *n* = 5).

Larvae of *E. tubifex* were isolated from *Triplophysa microphthalma* and identified morphologically [27]. Third-stage larvae were manually observed under a stereomicroscope, and their survival rate was assessed. Only batches with >95% viability were used for infection. Each duck in the infected group received 10–15 viable third-stage larvae (L3). Control ducks received an equivalent amount of sterile H_2_O and were fed without larvae. At 7 days post-infection, one duck from each group was humanely euthanized via approved methods for post mortem examination to confirm successful infection. Parasites visible on the proventriculus were counted under a dissecting microscope. The remaining ducks (*n* = 4 per group) were euthanized at 10 days for subsequent transcriptomic analysis. Parasites were computed as the total count of worms recovered per duck. No mortality occurred during the experiment. The proventriculus tissue was immediately collected. Tissue samples exhibiting nematode infection from the experimental group and corresponding healthy tissues from the control group were rapidly frozen in liquid nitrogen until subsequent RNA extraction.

### 2.2. RNA Sequencing, Bioinformatics, and Statistics

Total RNA was isolated from about 30 mg of ground proventriculus tissues at Shanghai Meiji Biomedical Technology Co., Ltd. (Shanghai, China). RNA content and purity were detected with NanoDrop 2000 (Thermo Fisher Scientific Inc., Waltham, MA, USA). RNA integrity was detected via agarose gel electrophoresis. The RQN was determined with Agilent 5300. A single library preparation requires a total RNA quantity of 0.5 μg, with a concentration ≥ 20 ng/μL, RQN > 4.5, and OD260/280 between 1.8 and 2.2.

Eukaryotic mRNA sequencing libraries were prepared by Shanghai Meiji Biomedical Technology Co., Ltd. Initially, mRNA was isolated from total RNA using magnetic beads coated with Oligo (dT) for A-T base pairing with polyA. The mRNA could be randomly broken into small fragments of about 300 bp after adding fragmentation buffer and selecting appropriate conditions. With mRNA as a template, one-strand cDNA was prepared via reverse transcriptase using random primers, and then stable two-strand cDNA was synthesized. The cDNA fragments underwent end-repair and adenylation. The products after adapter ligation were purified and sorted as fragments. The sorted products were PCR-amplificated and purified to from the final library. After USER enzyme digestion and PCR amplification, the resultant libraries were purified and subjected to quality assessment. Library quantification was conducted using a Qubit 4.0 fluorometer (Thermo Fisher Scientific Inc., Waltham, MA, USA). Sequencing was executed by an Illumina NovaSeq X plus platform with a paired-end read length of 150 base pairs.

The raw data from sequencing machines were uploaded to Majorbio Cloud (https://cloud.majorbio.com, accessed on 1 February 2026) for bioinformatics analysis. The analysis process primarily follows the description by Ren et al. [28]. The quality of the raw sequencing data was assessed on FastQC, and low-quality reads and adapter sequences were discarded with Trimmomatic to preserve high-quality clean data. After this preprocessing, the high-quality reads were mapped to the duck reference genome (specifically, the Anas platyrhynchos ASM874695v1 assembly available from NCBI) using the STAR aligner. To quantify transcript abundance, featureCounts was employed, and expression levels were computed as fragments per kilobase of transcript per million mapped reads (FPKM).

Differentially expressed genes (DEGs) between infected and control samples were identified with DESeq2 1.38.3 in the R statistical environment. Genes exhibiting an absolute log2 fold change (|log2FC|) exceeding 2 and an adjusted *p*-value (Padj) below 0.05 were deemed to show significant differential expression. The screened DEGs were submitted to Meiji BioCloud for functional annotation and enrichment analysis based on GO (http://geneontology.org/, accessed on 1 February 2026) and KEGG (https://www.genome.jp/kegg/, accessed on 1 February 2026) databases to identify significantly enriched biological processes and signaling pathways. The protein–protein interactions (PPIs) of the DEGs were examined with the STRING database (https://string-db.org/, accessed on 1 February 2026). A PPI network was built on STRING 12.0 (https://string-db.org/, accessed on 1 February 2026) with a confidence score threshold of 0.4. Hub genes were defined as nodes with a degree (number of directly connected interacting partners) in the top 10% of all nodes within the network, following established criteria for scale-free biological networks.

### 2.3. Validation by Quantitative Real-Time PCR (RT-qPCR)

To corroborate the RNA-Seq data, a subset of six DEGs exhibiting the highest and lowest expression levels (three with the most significant upregulation and three with the most significant downregulation) were chosen for independent validation using RT-qPCR. Primers specific to these targets were designed with the NCBI Primer-BLAST tool (https://www.ncbi.nlm.nih.gov/tools/primer-blast/, accessed on 15 August 2025), and their sequences are listed in Table 1. First-strand cDNA was synthesized from 1 µg of total RNA using a gDNA removal and reverse transcription kit (PrimeScript RT reagent kit with gDNA Eraser, Takara, Bio Inc., Kusatsu, Shiga, Japan). Each qRT-PCR reaction was carried out in triplicate for every biological sample. Reactions were assembled using a SYBR Green-based master mix (Premix Pro Taq HS qPCR kit, Accurate Biology, Hunan Acres Bio-engineering Co., Ltd., Changsha, China) and run on a QuantStudio 5 RT-PCR system (Applied Biosystems, Thermo Fisher Scientific, Foster City, CA, USA). The amplification protocol was enzyme activation at 94 °C for 30 s; 45 cycles of denaturation at 94 °C for 5 s, annealing at 55 °C for 15 s, and extension at 72 °C for 10 s. To confirm the absence of non-specific products or primer–dimer artifacts, a melting curve was plotted after the final cycle. The transcript levels of the target genes were normalized to the endogenous control, GAPDH. Relative changes in expression between the experimental groups were computed with the standard 2^−ΔΔCT^ method.

### 2.4. Transcriptome Profile Submission

The DEGs and raw files obtained from high-throughput sequencing were submitted to the NCBI Sequence Read Archive (SRA, PRJNA1359557).

## 3. Results

### 3.1. Confirmation of Infection and Evaluation of Transcriptome Sequencing

At 10 days post-infection, the necropsy of four ducks from the infected group demonstrated successful establishment of infection in three individuals, with a mean parasite burden of 5.3 ± 1.2 worms per duck (range: 4–7). The larvae viability ranged from 40% to 50%. No nematodes were detected in any of the examined ducks in the control group. Gross pathological examination of the proventriculus in the infected ducks showed varying degrees of hyperemia, multifocal hemorrhages, and mucosal thickening, whereas the control ducks exhibited no visible lesions.

We ensured the accuracy and relevance of the results, which guaranteed the sequencing results were statistically and biologically important. Every sample was tested in three biological repetitions, and 40.28 Gb of clean data were obtained. The raw sequencing data from each sample were deposited in the NCBI SRA under BioProject accession PRJNA1359557. Three samples from infected proventriculus tissues correspond to SRR36034978, SRR36034979 and SRR36034980, respectively, while three samples from uninfected proventriculus tissues are available under accession numbers SRR36034981, SRR36034982 and SRR36034983 respectively.

Quality control metrics for the Seq2Fun analysis are summarized in Table 2. Across all samples, the clean read rate (CRR) exceeded 99%, indicating that only a minimal proportion of reads were discarded due to low quality; a CRR above 90% is generally considered acceptable [24]. The proportion of reads successfully mapped to the reference genome was consistently above 65%, suggesting both the completeness of the annotation and the absence of significant contamination. For the cleaned data, the Q20 score was greater than 98% in every case, and the GC content ranged narrowly from 47.83% to 48.61%. As recommended by ENCODE guidelines, the squared Pearson’s correlation coefficient (R^2^) between replicates was above 0.92, confirming high reproducibility under the experimental conditions (Table 2).

**Figure 1:**
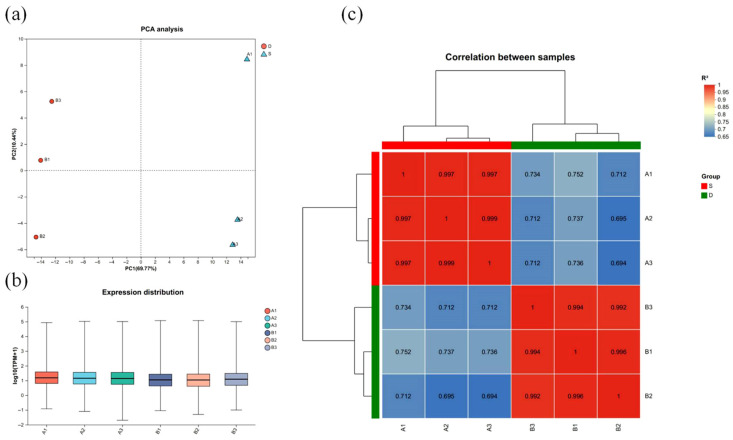
Evaluation of transcriptome sequencing data. (**a**): PCA of the sample (raw counts after normalization). (**b**): The horizontal axis is the sample name, and the vertical axis is log2 (FPKM + 1). The boxplot for each region stands for five statistics: maximum, upper quartile, median, lower quartile, and minimum from top to bottom. (**c**): Heatmap of correlations between samples. The horizontal and vertical axes in the graph are the squared of the correlation coefficient for each sample.

To visualize the global transcriptional patterns, a principal component analysis (PCA) was conducted with the normalized count data. The resulting plot (Figure 1a) shows a clear isolation between control and infected samples, with the infection status accounting for 69.77% of the total variance observed. The plot also reveals that while infected and control samples formed distinct clusters, there was comparable within-group variability in both conditions (Figure 1a). An examination of gene expression distributions (Figure 1b) showed that transcript levels were broadly comparable across both groups. Furthermore, a heatmap of sample-to-sample correlations (Figure 1c) demonstrated that all correlation coefficients exceeded 0.69. Collectively, these results confirm that the experimental manipulation introduced distinct and detectable transcriptional differences between the infected and control groups.

### 3.2. Analysis of DEGs

**Figure 2:**
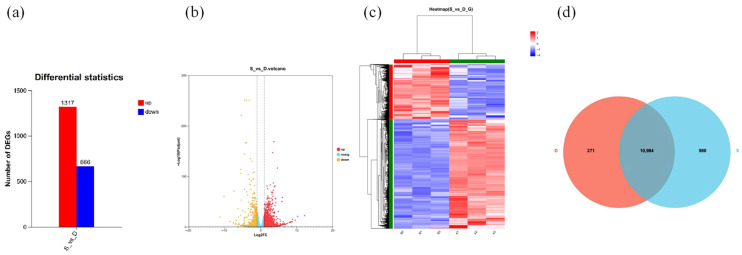
Differential gene expression analysis in domestic ducks *(Tadorna)* infected with *E. tubifex*. (**a**) Histogram summarizing the number of DEGs. Red and blue bars represent upregulated and downregulated DEGs, respectively. The number above each bar indicates the total count of DEGs. (**b**) Volcano plot of DEGs in the S_vs_D comparison group. The *x*-axis is the log2 fold change, and the *y*-axis shows −log10(Padj). The blue dotted lines indicate the threshold criteria for DEG screening (|log2FC| ≥ 2 and Padj < 0.05). Red and yellow dots denote significantly upregulated and downregulated genes, respectively. (**c**) Heatmap of DEG expression clustering. The *x*-axis shows sample names, and the *y*-axis is the normalized FPKMs of DEGs. Red and green indicate high and lower expression levels, respectively. (**d**) Venn diagram on the overlap of DEGs between groups S and D. Different colours represent distinct comparison combinations.

To investigate the gene expression changes in domestic ducks (*Tadorna*) infected with *E. tubifex*, the gene expression level was calculated based on the gene sequences in each sample using featureCounts, and FPKM was converted to obtain the normalized expression level. The gene expressions were quantitatively analyzed using RSEM. Differential expressions of the reference genes were analyzed with DESeq2, and DEGs with |log2FC| ≥ 2 and adjusted *p* < 0.05 were identified (Figure 2a). In the S_vs_D comparison group, a total of 1983 DEGs were screened, among which 1317 genes were upregulated, and 666 genes were downregulated (Figure 2a). A volcano plot was drawn to visualize the distribution of DEGs in the control group and show the statistical significance versus the magnitude of change in the expression of the transcripts. Even though the number of upregulated genes (1317) surpassed the number of downregulated genes (666), there were more significant transcripts with lower expression levels (log2FC > 2) than the ones with higher expression levels (log2FC < −2) (Figure 2b). DEGs in identical expression patterns across samples clustered. As shown in the heatmap (Figure 2c), the expression of DEGs was significantly altered in the S_vs_D comparison group. The overlap of DEGs among different comparison groups is illustrated using Venn diagrams, aiding in the identification of common or unique DEGs in specific comparisons. There were 271 common DEGs and 980 unique DEGs in the S group in samples under different infection conditions, while the D group contained 10,984 non-DEGs (Figure 2d). The results indicate that infection with *E. tubifex* induces significant dynamic changes in gene expression levels in domestic ducks.

### 3.3. GO and KEGG Pathway Enrichment Analysis

**Figure 3:**
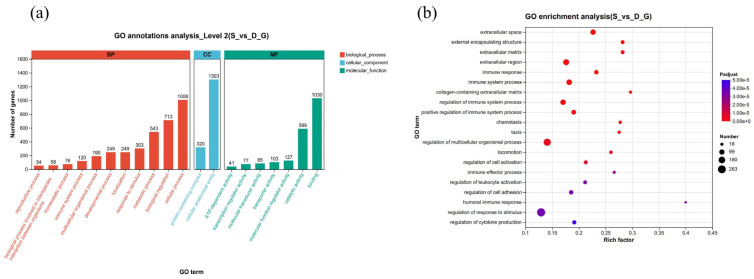
Gene ontology (GO) term enrichment analysis of differentially expressed genes in domestic ducks *(Tadorna)* infected with *E. tubifex*. (**a**) Bubble plot of GO enrichment analysis for the S_vs_D comparison group. The y-axis represents the GO terms, and the x-axis indicates the rich factor. Dot size corresponds to the number of genes assigned to each term, and color implies the significance of enrichment (−log10(*P*adjust)) (the same in Figure 4). (**b**) Level 2 GO classification histogram for the S_vs_D group. The x-axis is the number of genes, and the y-axis lists the GO terms grouped into BP, CC, and MF categories. Bars are colored according to the three major GO classification.

To detect the cellular response to *E. tubifex* infection in domestic ducks, we performed enrichment analysis to annotate gene functions and pathways. We used Goatools to conduct GO enrichment analysis on the DEGs from the S_vs_D comparison group and generated a bubble plot to visualize the enriched GO terms. Results showed that the DEGs were associated with three major categories: biological process (BP), cellular component (CC), and molecular function (MF). For GO functional enrichment, the threshold for significant enrichment was padj < 0.05. The DEGs were significantly enriched in CC terms such as extracellular space, external encapsulating structure, extracellular matrix (ECM), extracellular region, and collagen-containing ECM. BP terms included immune response, immune system process, regulation and positive regulation of immune system process, chemotaxis, taxis, locomotion, regulation of cell activation, immune effector process, regulation of leukocyte activation, regulation of cell adhesion, humoral immune response, regulation of response to stimulus, and regulation of cytokine production. MF terms included binding, catalytic activity and molecular function regulator. Additional MF subcategories significantly represented included transporter activity, molecular function transducer, reductase activity, and transcription regulator activity. A small number of genes were also annotated to structural molecule activity and molecular adaptor activity (Figure 3a).

We also generated a level 2 GO classification histogram, which displayed the number of genes annotated to each major functional category (Figure 3b). The results indicated widespread involvement of DEGs across multiple biological processes and cellular components.

**Figure 4:**
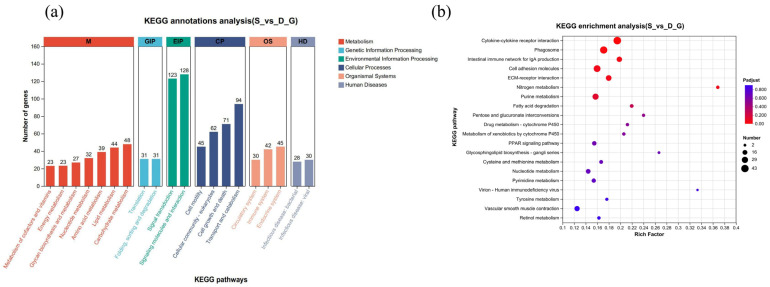
KEGG pathway enrichment analysis of DEGs in domestic ducks (*Tadorna*) infected with *E. tubifex*. (**a**) Bubble plot of KEGG enrichment analysis for the S_vs_D comparison group. The y-axis is the KEGG pathways, and the x-axis is the rich factor. (**b**) KEGG classification histogram for the S_vs_D group. The x-axis shows the number of genes, and the y-axis lists the KEGG pathways grouped into six major categories. Bars are colored according to the respective categories.

**Figure 5:**
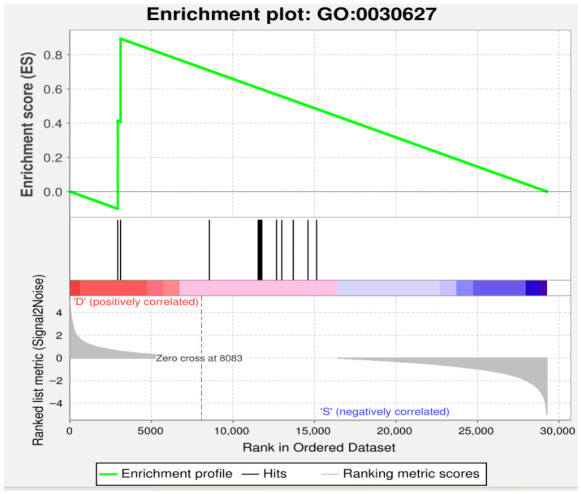
Gene set enrichment analysis (GSEA) of *E. tubifex* infection in domestic ducks (*Tadorna*). GSEA plot for gene set GO:0030627 in the S_vs_D comparison. The figure contains three main components: the top panel displays the enrichment score (ES) profile, where the peak ES implies the extent to which the gene set is over represented at the top or bottom of the ranking list; and the middle panel indicates the location of gene set members (“hits”) in the ranking list, showing where genes from the set appear along the ordered dataset; the bottom panel shows the distribution of the ranking metric (Signal2Noise) across all genes. The heatmap with the red region (“D”) indicates positive correlation with the infected group, and the blue region (“S”) indicates negative correlation. The signal-to-noise ratio for each gene (Signal2noise, based on the previously selected ranking metric) is shown as a gray-area plot. The vertical line marks the point at which the enrichment score reaches its maximum deviation (zero cross at 8083).

Genes within organisms do not operate alone but frequently engage in interactions with one another, leading to the formation of a variety of metabolic pathways and signal transduction networks. In this context, KEGG pathway enrichment analysis serves as a valuable tool for elucidating the biological functions of DEGs and for gaining insights into the biological systems of actively responding cells following nematode infection. The analysis was conducted using KOBAS v2.1.1 software (http://kobas.cbi.pku.edu.cn/download.php, accessed on 1 February 2026), which facilitated a comprehensive assessment of the involvement of the DEGs in relevant metabolic pathways. The KEGG enrichment analysis highlighted the identification of 20 significantly enriched pathways that play crucial roles in biological processes (Figure 4). Among these pathways are cytokine–cytokine receptor interaction, which is vital for immune responses; the phagosome pathway, significant for pathogen digestion; and the intestinal immune network for IgA generation, which is essential for gut immunity. Other important pathways revealed include those related to cell adhesion molecules, which facilitate cell interactions; ECM–receptor interaction, crucial for cellular communication; and the pathways involved in nitrogen metabolism and purine metabolism, both essential for cellular energy and nucleotide synthesis. Furthermore, pathways such as fatty acid degradation and drug metabolism via cytochrome P450 illustrate the capacity of the organism for processing and utilizing diverse substrates. Other notable pathways identified include the xenobiotics metabolism by cytochrome P450, the PPAR pathway, glycosphingolipid biosynthesis, cysteine and methionine metabolism, pyrimidine metabolism, and vascular smooth muscle contraction, all of which contribute to maintaining homeostasis and responding effectively to environmental challenges. Thus, this comprehensive analysis provides useful insights into the complicated interplay of metabolic and signaling networks following nematode infection (Figure 5).

**Figure 6:**
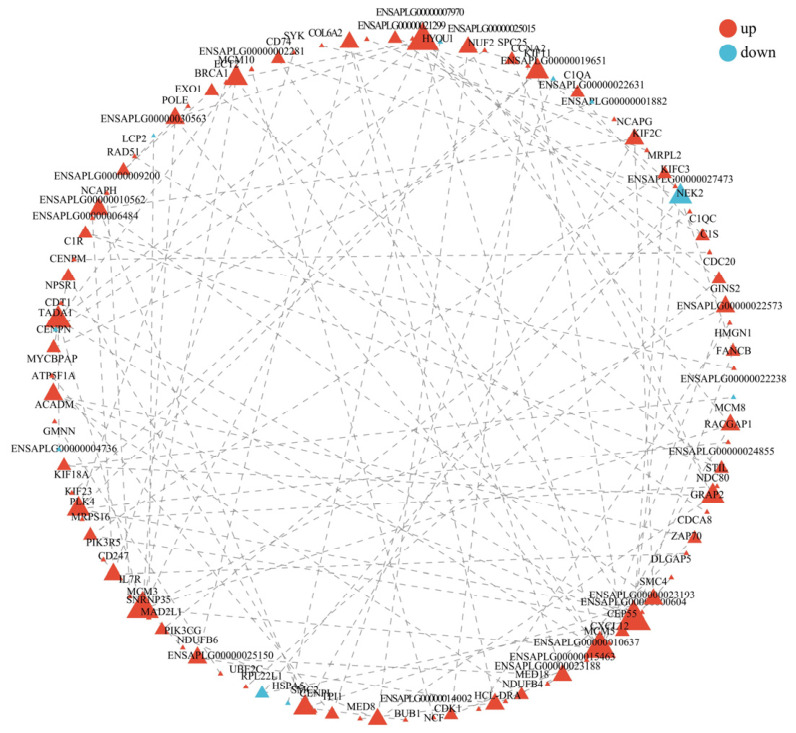
PPI network analysis of DEGs in domestic ducks infected with *E. tubifex*. The PPI network was built from DEGs in the S_vs_D comparison group using a threshold of |log2FC| ≥ 2 and *p* < 0.05. Nodes represent proteins encoded by DEGs; a bigger node means more connections (degree), so a highly linked gene shows up larger, hinting at its key role in the network. The dashed lines represent interactions between proteins.

Furthermore, KEGG classification demonstrated that the DEGs were distributed across six major categories: metabolism, genetic or environmental information processing, cellular processes, organismal systems, and human diseases. Within these categories, the DEGs were highly enriched in pathways associated with signal transduction, immune system, infectious diseases, carbohydrate metabolism, lipid metabolism, and amino acid metabolism (Figure 6).

These results indicated that the GO and KEGG pathway enrichment analyses identified multiple significantly enriched functional categories and signaling pathways, providing comprehensive insights into the molecular mechanisms of ducks in response to *E. tubifex* infection.

### 3.4. GSEA Enrichment Analysis

To avoid missing genes that were not significant DEGs but biologically important, we performed gene set enrichment analysis (GSEA). GSEA uses a preset gene set to rank the genes as per their differential expression levels in two classes of samples and then checks if the preset gene set is enriched at bottom or top of the ranking list. The analysis was conducted between the control and infected groups (S_vs_D).

The gene set GO:0030627 was significantly enriched in the infected group. The enrichment score (ES) reached approximately 0.8, indicating a strong positive correlation with the infection phenotype. The zero cross point occurred at rank 8083 in the ordered dataset, and the gene set was prominently enriched among genes positively correlated with infection (“D” group). The enrichment profile and ranking metric (Signal2Noise) further confirmed that this biological process was systematically activated upon *E. tubifex* infection (Figure 5).

These findings suggest that the GO term GO:0030627, though not individually prominent in differential expression analysis, plays a functionally coordinated role in the host response to nematode infection, highlighting the value of GSEA in uncovering biologically meaningful gene sets beyond conventional DEG screening.

### 3.5. PPI Network Analysis of DEGs

To probe the biological relevance of the DEGs, we performed PPI network analysis of the DEGs using STRING 12.0. The PPI network was constructed and visualized based on DEGs with FDR ≤ 0.05 and |Log2FC| ≥ 2 from the S_vs_D comparison group.

The PPI network analysis revealed a complex interaction network among the DEGs associated with *E. tubifex* infection in domestic ducks (*Tadorna*). The network contained multiple highly connected modules, and some nodes exhibited high betweenness centrality (BASAL score), indicating their key roles in maintaining network stability and signal transmission. These hub genes may be critical in regulating host immune responses, ECM organization, and metabolic adaptation during nematode infection.

The results indicate that the identified hub genes may function coordinately in multiple biological processes, including immune activation, cell adhesion, chemotaxis, and metabolic adjustment, suggesting their potential importance in mediating the host–pathogen interaction and the pathological progression of nematode infection (Figure 6).

### 3.6. Validation of DEGs by qPCR

**Figure 7:**
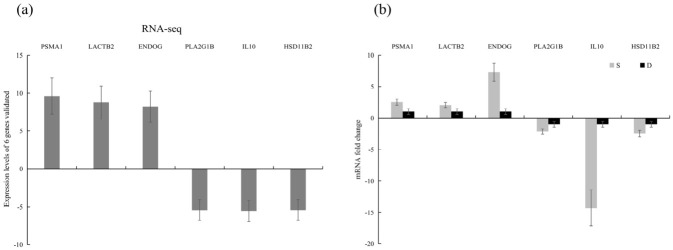
Relative expression of DEGs validated by RT-qPCR in domestic ducks infected with *E. tubifex*. (**a**) Verification of expressions levels of six genes randomly chosen by RT-qPCR. With the GAPDH gene as the internal reference, the relative expression (fold change) of each gene was determined by comparing the 2^−ΔΔCT^ method. Values (RT from qPCR) are reported as mean ± SD. N = 3. (**b**) Expression fold changes at six randomly selected levels based on RNA-seq transcriptome analysis of duck samples. Data are reported as log2 (fold change) and mean ± SD. N = 3.

To verify the reliability and reproducibility of the DEGs screened from transcriptome sequencing, we randomly chose six genes for qPCR: PSMA1, LACTB2, ENDOG, PLA2G1B, IL10, and HSD11B2. The relative expressions levels of these genes significantly differed between the experimental and control groups (Figure 7a). In the experimental group (*E. tubifex*-infected domestic ducks (*Tadorna*)), FSMA1, LACTB2, ENDOG, and PLACGB were markedly upregulated, while HL16 and HSD1-R2 were downregulated. Figure 7b compares the mRNA fold changes obtained from qPCR and transcriptome sequencing. The expression trends of all six genes are consistent between the two methods, confirming that the DEGs identified by RNA-Seq are reproducible and reliable. These qPCR results demonstrate that the transcriptome sequencing data accurately reflect the expression changes of key genes in response to *E. tubifex* infection, thereby validating the high repeatability and credibility of our sequencing analysis.

## 4. Discussion

The present study provides a comprehensive transcriptomic analysis of the molecular responses in domestic ducks infected with *E. tubifex*, revealing significant alterations in gene expression profiles, enriched biological pathways, and complex PPI networks. Our findings not only corroborate the expected activation of immune-related processes but also highlight nuanced metabolic adaptations and potential key regulators in the host–parasite interaction. The robustness of our sequencing data, validated by qPCR, offers a reliable foundation for interpreting the molecular mechanisms underlying this infection.

High-quality transcriptome data, with high mapping rates and reproducibility among biological replicates, provide reliable basis for downstream analysis [29]. Large amounts of transcriptional reprogramming (over 1983 DEGs) suggest that the host has an active response to the parasite and repairs tissue damage, as supported by GO and KEGG enrichment analyses. Rather than operating in isolation, the enriched immune and tissue remodeling pathways appear to form an integrated response network. Large amounts of immune terms such as “immune response”, “immune system process” and “chemotaxis”, along with pathways such as “cytokine–cytokine receptor interaction” and “phagosome”, suggest classical defense against helminth infections [20,21,30,31]. This immune activation is complemented by parallel upregulation of pathways engaged in ECM organization and cell adhesion molecules, which likely serve dual functions: repairing physical damage inflicted by the parasite and facilitating the recruitment and anchoring of immune cells to infection sites [32,33,34]. At the signaling level, the concurrent activation of NOD-like receptor and toll-like receptor pathways [35], both critical for recognizing pathogen-associated molecular patterns (PAMPs) and starting innate immunity, further supports a coordinated frontline defense. Their activation in our model is consistent with results in other avian–helminth systems where receptors trigger downstream cascades leading to production of proinflammatory cytokines and chemokines and recruitment and stimulation of immune cells such as macrophages and heterophils [36,37]. Furthermore, enrichment of “antigen processing and presentation” highlights innate and adaptive immunity, suggesting that some T cells are preparing for nematode defense [38,39]. Together, these findings suggest that the duck gastric mucosa mounts a multi-layered functionally integrated response to nematode infection, encompassing pathogen sensing, inflammatory cell recruitment, tissue repair, and adaptive immune priming.

Beyond the DEGs, the observed transcriptomic landscape likely reflects the activity of upstream regulatory networks. RNA-binding proteins (RBPs), particularly those containing RNA recognition motifs such as RBMX2, have appeared as critical post-transcriptional regulators of immune responses and tissue remodeling [40]. In helminth infections, RBPs can modulate mRNA stability and splicing of cytokines, chemokines, and extracellular matrix components, thereby fine-tuning the host response [40]. Notably, our study parallels the transcriptomic changes observed following *Mycobacterium bovis* infection, in which the protein RBMX2 is identified as a key host factor that modulates apoptosis via APAF-1 alternative splicing and facilitates pathogen invasion by compromising tight junction integrity [41,42]. Although RBMX2 was not among the most significantly upregulated genes in our dataset, its moderate yet consistent expression change suggests it might contribute to post-transcriptional regulation during gastric nematode infection, warranting further functional investigation of RBMX2 homologs in the duck genome.

Similarly, transcriptional regulators including members of the NF-κB, STAT, and interferon regulatory factor (IRF) families can orchestrate anti-helminth immunity [43,44]. The enrichment of immune-related pathways observed in our GO analysis (e.g., ‘regulation of immune system process’ and ‘regulation of cytokine production’) likely reflects the downstream effects of these upstream regulators. Future research using chromatin immunoprecipitation sequencing (ChIP-seq) or targeted perturbation experiments would help to identify the specific transcription factors driving the observed expression patterns.

One interesting finding is the enrichment of the metabolic pathway “intestinal immune network for IgA production”. IgA plays an important role in mucosal immunity, and its induction is common in gastrointestinal nematode infections across species [45]. Moreover, enrichment of metabolic pathways including “Fatty acid degradation”, “Purine metabolism”, and “Pyrimidine metabolism” indicates that the infected host is changing metabolically and thus provides energy for the demanding immune response [46]. Downregulation of the “PPAR signaling pathway”, a key regulator of lipid metabolism and inflammation, can further support metabolic reprogramming and may suggest a mechanism for modulating the immune response [47], suggesting a potential mechanism for immunosuppression observed in chronic helminth infections, as PPAR signaling has anti-inflammatory effects.

The PPI network identified hub genes with high betweenness centrality, including IL6, STAT3, FOS, and JUN, which implies that they coordinate the host response. The centrality of IL6 and STAT3 strongly reflects the IL-17 pathway and Th17-type responses, which are increasingly recognized to be important in mucosal immunity against extracellular pathogens, such as helminths [25,48,49,50]. Coordinated upregulation of FOS and JUN (parts of AP-1 transcription factor complex) suggests that the cell processes such as proliferation, differentiation and apoptosis are activated together due to tissue injury and inflammation at the infection site. Genes central to immune signaling (e.g., cytokines, receptors) and tissue integrity are likely among these hubs orchestrating the complex interplay of inflammation, tissue repair, and metabolic adaptation. This network approach provides a system view, suggesting that host response is not merely a set of individual gene changes but a highly connected functional module [49,51]. The PPI network was constructed based on computationally predicted interactions from the STRING database, which extrapolates from model organisms rather than duck-specific experimental data. Consequently, the identified hub genes should be considered as candidates requiring experimental validation through targeted approaches, such as co-immunoprecipitation or yeast two-hybrid assays.

Our GSEA results further refine this picture by finding coordinated biological process (GO:0030627), which, although not individually important in DEG analysis, was activated after infection. This shows how GSEA can be useful for discovering small but biologically coherent changes that could not be detected by threshold methods [52].

Compared to the transcriptome of other animals with intestinal helminths (Chickens with *Ascaridia galli* or *Heterakis gallinarum*), we find the same themes of activated innate immunity, cytokine signaling and mucosal defense [53,54]. Strong induction of TLR and cytokine signaling pathways has been reported for chickens with *Ascaridia galli* or *Eimeria* spp. However, differences may arise in the specific metabolic adaptations. Some studies in mammals show a shift towards Th2-polarized immunity with alternative macrophage activation [55,56,57], but we found a more complex strong inflammatory (Th1/Th17-skewed) response and metabolic suppression simultaneously in domestic ducks. This difference may arise from the particular host species, nematode, or time point post-infection that is chosen [58]. Species-specific differences (e.g., the number of enriched metabolic pathways and the key hub genes in the PPI network) probably depend on host species, specific nematode parasite, and tissue tropism and shall therefore be studied [58,59,60].

However, our results have limitations. First, the transcriptomic data are only available after infection. The host–parasite interaction is dynamic. Time-course experiments would be needed to distinguish early defense mechanisms from later adaptation or pathology. Second, bulk gastric tissue (i.e., epithelium, immune cells, fibroblasts) was sampled, and the gene expression profiles represent average signals. In addition, the within-group transcriptional variability is likely due to the combined effects of subclinical co-infections, genetic and microbiota differences, and the limited sample size amplifying individual variation. Important cell type responses could be hidden by the presence of epithelial cells and/or infiltrating lymphocytes. Third, bioinformatic predictions are powerful, but functional validation is required. Future research should focus on time profiling to capture the host response dynamics. Functions of hub genes from the PPI network and key pathways (e.g., PPAR signaling) would also require experimental validation using knockdown/overexpression studies or in vitro drug therapy to identify their role in host defense or pathology.

Finally, we show how a complex transcriptional network characterized by domestic ducks infected by *E*. *tubifex*, which describes a strong and diverse transcriptional response in the duck proventriculus following *E*. *tubifex* infection, can be used to understand how waterfowl–helminth interactions interact; we also show how this network can be used to design functional studies to develop new control strategies.

## 5. Conclusions

This paper provides a transcriptomic analysis of the domestic duck response to *E*. *tubifex* infection. The data show that infection triggers a coordinated immune response with upregulation of genes involved in immune defense, chemotaxis, extracellular matrix remodeling and cytokine signaling. An enrichment of pathways involving phagosome activity, leukocyte activity, and cell adhesion underscores that host defense has to be aimed at limiting parasitic invasion and tissue damage. Several genes have emerged as potential hubs in a protein–protein interaction network, but these observations are correlative and require functional testing.

## Figures and Tables

**Table 1:** Primers for RT-qPCR.

Gene Name	Primers	Primer Sequence (5′-3′)
PSMA1	PSMA1-F PSMA1-R	GCGTGCTCTCAGAGAGACTC CAGGTTTCAGTCCCAGCCAT
LACTB2	LACTB2-F LACTB2-R	AAATGAGTGCTGAGCTCCCC GCACACCACCATTCAAACCC
ENDOG	ENDOG-F ENDOG-R	GGACCCCAGTCAGAACAGTG AGCAGCAGAACCCATGAGAC
PLA2G1B	PLA2G1B-F PLA2G1B-R	CCGTTGTGCTTCTCCTCAGT GCAGCCGTAGCCATCATACT
IL10	IL10-F IL10-R	CGGGGTGGTGTGGTTAATGA GCACCCTTCGGTTTCTAGCT
HSD11B2	HSD11B2-F HSD11B2-R	AGGAGAGGACCAGAATCGCT GAACACGTTCAGCCTCCTGA

**Table 2:** Transcriptomic sequencing data.

Sample	Raw Reads	Clean Reads	CRR(%)	Mapped Reads	Total Mapped Reads (%)	GC(%)	Q20(%)	Q30(%)
A1	49,891,230	49,575,710	99.37	39,811,446	39,811,446 (80.3)	48.12	99.00	96.77
A2	42,876,342	42,581,840	99.31	33,335,913	33,335,913 (78.3)	48.71	98.95	96.59
A3	42,874,500	42,576,512	99.30	33,510,773	33,510,773 (78.7)	48.65	98.96	96.62
B1	48,259,708	47,959,008	99.38	39,954,866	39,954,866 (83.3)	48.42	98.99	96.72
B2	43,498,202	43,240,930	99.41	35,870,604	35,870,604 (82.9)	47.88	99.04	96.87
B3	42,707,296	42,461,350	99.42	35,653,525	35,653,525 (84.0)	47.83	98.97	96.62

Note: CRR: clean read rate; A1, A2 and A3 correspond to the infected group; B1, B2 and B3 samples correspond to the control group.

## Data Availability

The original data presented in the study are openly available in the NCBI Sequence Read Archive (SRA) under BioProject accession number PRJNA1359557.

## Abbreviations

RNA-seq	RNA sequencing
DEGs	Differentially expressed genes
FPKM	Fragments per kilobase of transcript per million mapped reads
GSEA	Gene set enrichment analysis
PCA	Principal component analysis
GO	Gene ontology
KEGG	Kyoto Encyclopedia of Genes and Genomes
